# Designing casein-coated iron oxide nanostructures (CCIONPs) as superparamagnetic core–shell carriers for magnetic drug targeting

**DOI:** 10.1007/s40204-014-0035-6

**Published:** 2014-12-19

**Authors:** Anamika Singh, Jaya Bajpai, Atul Tiwari, Anil Kumar Bajpai

**Affiliations:** 1 BMRL, Govt. Model Science College, Jabalpur, India; 2 Mechanical Engineering, University of Hawaii at Manoa, 2540 Dole Street, Holmes #302, Honolulu, HI 96822 USA

**Keywords:** Nanoparticles, Casein, Casein-coated iron oxide nanoparticles, Characterizations, Superparamagnetic, Magnetic drug delivery

## Abstract

Magnetic drug targeting is a drug delivery system applicable to cancer treatment. Coated magnetic particles, called carriers, are very useful for delivering chemotherapeutic drugs. In the present research, casein-coated iron oxide nanocarriers (CCIONPs) of core shell nanostructure have been described as being applicable to magnetic drug targeting. The structure, morphology, and composition of prepared magnetic nanoparticles were determined by analytical techniques like Fourier transform infrared spectroscopy (FTIR), transmission electron microscopy (TEM), scanning electron microscopy (SEM), Electron diffraction (ED), X-ray diffraction (XRD), Zeta potential, Dynamic light scattering (DLS), Mossbauer and Raman spectroscopy, X-ray photoelectron spectroscopy (XPS), Vibrating sample magnetometery (VSM)) and in vitro cytotoxicity analysis. Magnetization studies of CCIONPs conducted at room temperature using a vibrating sample magnetometer suggested their superparamagnetic nature as having a saturation magnetization (Ms) of 64 emu g^−1^ at an applied magnetic field of 5 kOe. The size of the magnetic polymeric nanoparticles was found to lie in the range of 73.9 ±0.36 nm, and the particles exhibited superparamagnetic behavior. The prepared particles could be used as a drug carrier for controlled and targeted drug delivery.

## Introduction

The srtudy of submicron magnetic entities is exciting and they have been extensively researched and employed in biomedical applications such as targeted drug delivery, magnetic resonance imaging, hyperthermia, etc. (Gyergyek et al. 2008). Although iron-based oxide materials like magnetite and maghemite have been commonly used in pharmaceutical and biomedical fields, cobalt- and platinum-based oxides either alone or in combination with iron have also been investigated. In biological applications, the particle surface is normally exposed to physiological environments and would be oxidized if bare particles are directly used. Apart from this, the bare particles are very sensitive to agglomeration to produce a large cluster due to their large surface to volume ratio and dipole–dipole interactions. All these undesirable consequences damage magnetic structures, and properties as well as their end performance. Thus, prior to using these metallic nanoparticles for human body applications, the aspects of their surface modification must be addressed. It is worth mentioning here that a rational modification of a nanoparticle's surfaces not only prevents agglomeration but also guarantees its enhanced biocompatibility, water solubility, and non-specific adsorption to cells (Indira et al. 2010).

There are numerous strategies to modifying nanoparticle surfaces for better performance and tailor made applications. The most common methods include adsorption onto nanoparticle surfaces, the addition of a second layer, the post addition of water soluble ligands, and the coating of functional materials to yield organic–inorganic hybrids, etc. Among all these approaches, coating of iron oxide nanoparticles with synthetic or natural polymers is the most effective at inducing surface modification of nanomaterials so as to transform them into core–shell nanostructures having metal oxide as the core and an organic polymer layer as the shell (Oh et al. 2011).

In magnetic core/shell type materials, the magnetic core is coated with a layer of nonmagnetic polymeric material. The inorganic core, when used alone, does not have a specific target, however, if the layer is a particular molecular precursor or is conjugated to a specific molecule, it can direct the particle to an area of interest (Jung et al. 2011). In these types of nanomaterials, the polymeric layer provides the interface between the core and the surrounding environment and can serve two purposes. Firstly, it acts as a barrier between the nanoparticle core and the environment, to protect and stabilise the core. Secondly, the chemical nature of polymeric layers dictates the reactivity, solubility, and interfacial interactions of the nanoparticle, and may also determine the biological handling. The hybrid nature provides a high chemical specificity that has been used to tailor materials with desirable functionalities (Yang et al. 2006). They can also be expected to facilitate intravenous delivery of drugs to desired sites (e.g., a tumor) using an external magnetic field. This approach is called magnetic drug targeting (MDT) and has emerged as a versatile technique for treating complex diseases. By this technique, high concentrations of chemotherapeutic or radiological agents can be achieved near the target site without any toxic effects to normal surrounding tissue. (Chomoucka et al. 2010).

Casein, a major milk protein, comprises about 94 % protein and 6 % low molecular weight compounds collectively called colloidal calcium phosphate. Four casein phosphoproteins, αS1-, αS2-, β-, and k-casein, in approximate proportions of 4:1:4:1 by weight, respectively, comprised the colloid. Their molecular weights are between 19 and 25 kDa and their average isoelectric point is between 4.6 and 4.8 (Fox et al. 2003; Thomsan et al. 2009). Casein has a strongly acidic peptide of 40 amino acids that contains 7 of the 8 phosphate groups, 12 carboxyl groups and only 4 positive groups. The highly charged N-terminal region of β-casein contains four of the five phosphates of the molecule, seven carboxyl groups and only two positive groups (Phandungath et al. 2005; Dalgeish et al. 1998).

Looking to the multifunctional structure profile of caseins, the present study adopts a novel strategy for preparation of superparamagnetic core/shell type nanoparticles, in which casein has been employed for coating of the iron oxide nanoparticles, crosslinked with glutaraldehyde by using an in situ co-precipitation method. Furthermore, the morphological, magnetic, and structural properties of so prepared nanoparticles have also been investigated by techniques like TEM, VSM, Raman and Mossbauer spectrometry.

### Experimental details

#### Materials

Casein (M.W. 22,068-23,724) was purchased from Merck, Mumbai, India and used without any pretreatment. FeCl_2_·H_2_O, FeCl_3_·6H_2_O, and glutaraldehyde (used as a crosslinker) were obtained from Loba Chemie, Mumbai, India. Toluene obtained from Sigma-Aldrich Corp. (St. Louis, MO, USA) was used for preparing the oil phase. Other chemicals like acetone, NaOH, etc. used were of standard quality (AR) grade. Double distilled water was used throughout the experiments.

### Preparation of casein-coated iron oxide nanoparticles (CCIONPs)

Preparation of magnetic casein nanoparticles involves a two step process in which the first step involves the preparation of casein nanoparticles (CNPs) and in the second step impregnation of iron oxide within the casein nanoparticles matrix.

#### Preparation of casein nanoparticles

In order to prepare casein nanoparticles, the microemulsion method was adopted as described in literature (Bouchemal et al. 2004; Jahanshahi et al. 2008). An aqueous phase was prepared by dissolving a known amount of casein in 1 % alkaline solution of NaOH, while for preparing the ‘oil phase,’ toluene was used. The above two solutions were mixed with vigorous shaking (shaking speed 1,000 RPM, 5 L capacity, Remi, India) for 30 min and to this emulsion 1 mL of glutaraldehyde was added as a crosslinker with constant stirring to take place for 30 min at room temperature (30 °C), then H_2_SO_4_ was added to the solution for the solidification of particles. The nanoparticles were cleaned by washing them thrice with acetone and the prepared casein nanoparticles were stored in air-tight polyethylene bags.

#### Reaction mechanism of the preparation of CNPs

**Fig. 1:**
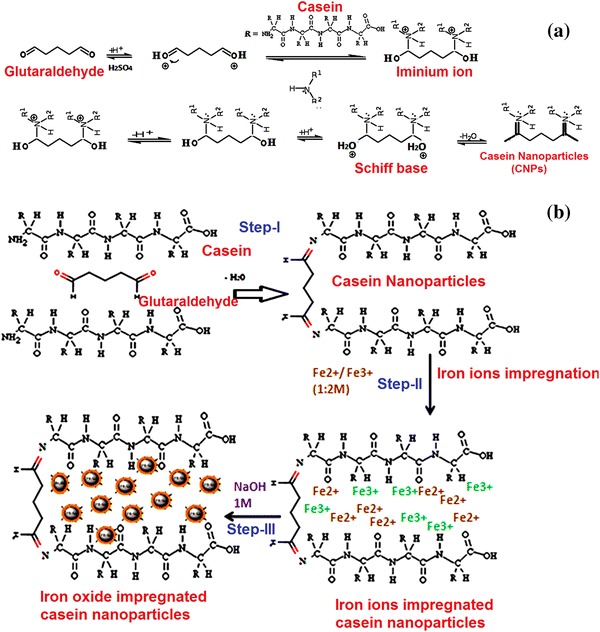
**a** Reaction mechanism of the preparation of CNPs, **b** Reaction scheme of the preparation of CCIONPs

In the preparation of CNPs, glutaraldehyde reacts with casein protein in acidic conditions (pH 1–2). The aldehyde group of glutaraldehyde can combine with nitrogen and some other atoms of proteins, or with two such atoms if they are very close together, forming a cross-link –CH_2_– called a methylene bridge. All these –CHO groups will combine with protein nitrogens with which they come into contact, so there is enormous potential for cross-linking. The formation of CNPs is an example of nucleophilic addition of an amino group to a carbonyl of glutaraldehyde followed by dehydration of the Schiff base (Migneault et al. 2004). The Schiff base is an electrophile that reacts in the second step in an electrophilic addition with a compound containing an acidic proton. This type of reaction is also considered a condensation reaction. The reaction mechanism of the preparation of CNPs is shown in Fig. 1a.

#### Impregnation of Iron oxide into the casein nanoparticles

The most common synthesis route to produce magnetite (Fe_3_O_4_) is the co-precipitation of divalent and trivalent iron from the respective hydrated salts in an alkaline medium (Lu et al. 2007; Wan et al. 2007). The dried nanoparticles were placed in an aqueous mixture of Fe^2+^ and Fe^3+^ chloride salts at a 1:2 molar ratio and allowed to swell for 24 h so that both Fe^2+^ and Fe^3+^ ions were entrapped into the biopolymer matrices (casein). Prior to putting them in salt solution, a dry stream of N_2_ was flushed for at least 15 min to control the reaction kinetics, which are strongly related with the oxidation speed of the iron species. Bubbling nitrogen gas through the solution not only protects critical oxidation of the magnetite but also reduces the particle size (Kim et al. 2001). After that, an alkaline solution of NaOH was added for a definite time period so that Fe^2+^ and Fe^3+^ ions were precipitated to the polymer matrix and magnetite was obtained per the following chemical reaction.

1 $${\text{Fe}}^{2+}+\;2{\text{Fe}}^{3+}+\;8{\text{OH}}^{-}⟶{\text{Fe}}_{3}{\text{O}}_{4}+\;4{\text{H}}_{2}\text{O}$$

Care should be taken while adding NaOH because, according to the thermodynamics of this reaction, a complete precipitation of Fe_3_O_4_ occurs in the range of 9 to 14 pH, and in a molar ratio of 1:2 for Fe^2+^:Fe^3+^ under a non oxidizing oxygen free environment. Otherwise, Fe_3_O_4_ might get oxidized as the following.

2 $${\text{Fe}}_{3}{\text{O}}_{4}+\;0.25{\text{O}}_{2}+\;4.5{\text{H}}_{2}\text{O}\;⟶3\text{Fe}{\left(\text{OH}\right)}_{3}$$

The change in color of the casein nanoparticles from orange to dark brown also confirms formation of iron oxides. The prepared nanoparticles were then washed, dried at room temperature and stored in airtight polyethylene bags. The percentage impregnation of iron oxide can be calculated using following equation (Gupta et al. 2014a, b).

3 $$\text{Impregnation}\;\;\left(\%\right)=\frac{W_{\text{impregnated}}-W_{\text{dry}}}{W_{\text{dry}}}\times 100$$

In this method, the nanoparticles of metal and metal oxides are synthesized within a pre-formed polymer frame work or matrix, in which the metal ion or the metal-precursor is first adsorbed on the polymeric host, followed by either a change in the valence state of the metal ion (reduction/oxidation) or in situ precipitation. The most important factors that affect the properties of CCIONPs are as follows:

(i) nature of the functional polymer and concentration
(ii) crosslinker type and concentration
(iii) type of nanoparticle pre-cursor
(iv) amount and type of the surfactant used
(v) reaction that forms the nanoparticles
(vi) composition of the metal and metal oxide nanoparticles.
(vii) Stirrer-reactor geometry and the rate of stirring (Ismail et al. 2013).

In this process, the polymers serve as nano-reactors and provide a confined medium for synthesis; also, they stabilize and isolate the synthesized nanoparticles preventing their aggregation. Although boundaries between the different processes of synthesis are very tight, the in situ process can be subdivided into two groups, namely, (i) sorption followed by a redox and/or precipitation reaction, and (ii) impregnation followed by a precipitation and/or redox reaction. Sorption is followed by an in situ redox and/or precipitation reaction (Sarkar et al. 2012). In impregnation of the metal ion or the metal precursor does not involve any chemical interaction between the functional groups of the functional polymer, the metal ions, or a metal precursor. In this case, the polymeric matrix limits the growth of magnetic particles; as a result. their size becomes smaller than in the absence of a polymer. The reaction scheme is shown in Fig. 1b.

## Characterization

### Fourier transform infra red analysis

The Fourier transform infrared (FTIR) spectra of casein and CCIONPs were recorded on an FTIR-8400 Shimadzu spectrophotometer. Samples for the spectral analysis were prepared by mixing nanoparticles and KBr in a 1:10 proportion and the spectra were obtained in the range of 4,000 to 400 cm^−1^ with a resolution of 2 cm^−1^.

### Scanning electron microscope analysis

Morphological studies of cross-linked CNPs and CCIONPs were performed using scanning electron microscopy (SEM, Philips 515, fine coater, Philips, Eindhoven, The Netherlands). Drops of the polymeric nanoparticles suspension were placed on a graphite surface and freeze-dried. The sample was then coated with gold by ion sputter at 20 mA for 4 min, and observations were made at 10 kV.

### Transmission electron microscope analysis

The size and morphology of the nanoparticles were determined by conducting transmission electron microscopy (TEM) analysis of casein and CCIONPs using a Morgagni 268-D transmission electron microscope with an accelerating voltage of 80.0 kV.

### Electron diffraction analysis

In order to investigate the crystalline nature of prepared casein and the electron diffraction (ED) of the iron oxide-impregnated nanoparticles, studies were performed using a Morgagni 268-D transmission rlectron microscope. The polycrystallinity of the samples is evident from the presence of rings. The *d* spacings are calculated from the diffraction patterns with the following relationship (Zou et al. 2011):

4 λL=dR

where*λ* is the electron wavelength,*d* is the lattice parameter, and R is the radius of the rings.

### Dynamic light scattering measurements

Dynamic light scattering (DLS) investigation of nanoparticles was carried out on a Delsa™ Nano Beckman Coulter instrument, which provides information about the size of the prepared particles. The Stokes–Einstein equation may be used to determine the hydrodynamic radius of particles in solution (Hoo et al. 2008), as follows:

5 $$D=\frac{kT}{6\pi hRn}$$

where *D* is the diffusion coefficient, *k* is Boltzmann’s constant, *T* is the temperature, *η* is the solvent viscosity, and *R*_h_ is the hydrodynamic radius of the particles in solution.

### Zeta potential measurements

In order to understand the nature of the particles and the prevailing interaction between the iron oxide and casein nanoparticles, surface potential studies were performed with a Beckman Coulter Delso Nano C instrument.

### X-ray diffraction analysis

X-ray diffraction studies of the nanoparticles were carried out on a D8 Advance, X-ray powder diffractometer. The diffraction data were collected from 10 to 70°, 2*θ* values with a step size of 0.02°, and a counting time of 2 s/step. The amorphous and crystalline nature of the casein nanoparticles and casein-coated iron oxide nanoparticles are also quantified in terms of degree of crystallinity calculated using the following equation (Singh et al. 2014):

6 $$X_{c}\left(\%\right)=\frac{A_{c}}{A_{a}+A_{c}}\times 100$$

where *A*_c_ and *A*_a_ are the areas of crystalline and amorphous phases, respectively. The average crystallite size of iron oxide particles were estimated using Scherer’s formula (Likhitkar et al. 2013) as follows:

7 d=kλ/βcosθ

where *d* is the mean grain size, *k* is the shape factor (0.9), *β* is the broadening of the diffraction angle, and *λ* is the diffraction wavelength (1.54 Å).

### X-ray photoelectron spectral (XPS) analysis

The samples were also analyzed by X-ray photoelectron spectroscopy (±) on a modified laser ablation system (Riber LDM-32) using a Cameca Mac3 analyzer. Photoelectron spectra were collected by acquiring data for every 1.0 eV with an energy resolution of 3 eV. Narrow-scan photoelectron spectra were recorded for C 1*s*, N 1*s*, O 1*s*, and Fe 2*p* by acquiring data for every 0.2 eV and the energy resolution was 0.8 eV.

### Raman spectral analysis

In order to investigate the impregnation of iron oxide nanoparticles into the matrix of casein nanoparticles, Raman spectroscopy was used and the spectra were obtained in the range of 200–1,800 cm^−1^. The characteristic peak position of magnetite (Fe_3_O_4_) and its possible oxidation products maghemite (У-Fe_2_O_3_) and hematite (a-Fe_2_O_3_) were determined in the Raman region of 100–1,200 cm^−1^. The Raman spectra of casein and casein magnetic nanoparticles were recorded on a Micro Raman Spectrometer (Jobin–Yvon Horibra LABRAM-HR).

### Mossbauer spectral analysis

The superparamagnetic behavior of magnetic nanoparticles was determined with a Mossbauer spectrophotometer at definitive temperatures.

### Vibrating sample magnetometer

The magnetization versus magnetic field plot (M–H first magnetization curve and hysteresis loop) at 300 K for the ferrite nanoparticles (powder sample) was measured by a 14T PPMS-vibrating sample magnetometer.

### In vitro cytotoxicity test

In order to determine the biocompatible nature of the prepared materials, a test on the extraction method (ISO10993-5, 2009) was applied as described here. In this method, powdered (0.2 g) material was soaked in culture medium (1 mL) with serum and then the extract was prepared by incubating the presoaked test material with the serum for 24 h. After incubation, the extract was filtered using 0.22 µm millex gp filter. 100 % extract were diluted with culture medium to get 50 and 25 % concentrations. Different dilutions of test sample extracts, positive control, and 100 % extracts of negative control in triplicate were placed on a subconfluent monolayer of L-929 cells. After incubation of cells with extracts of the test sample and controls at 37 ±1 °C for 24 to 26 h, the culture was examined microscopically for cellular response. For negative control, the sample was prepared by incubating a 1.25 cm^2^ polyethylene disc with 1 mL of culture medium with serum at 37 ±1 °C; a positive control was prepared by diluting phenol stock solution (13 mg/ml) with culture medium with serum (Shi et al. 2010).

### Statistical analysis

All experiments were done at least thrice and figures and data have been expressed along with the respective error bars and standard deviations, respectively.

## Results and discussion

### Effect of iron salts on iron oxide impregnation

In the present study, an in situ coprecipitation method was used to design CCIONPs, as discussed under the methods section. Inclusion of iron oxide into the polymer matrix results from in situ precipitation of ferrous/ferric ions when treated with alkaline solution. In order to study the effect of composition of iron oxide in CNPs, the CCIONPs containing different iron oxides were prepared by changing the oxidation form of iron as well as its molar ratio. In these experiments, the CNPs were allowed to swell in three different iron salt solutions viz. Fe^2+^ (0.5 M), Fe^3+^ (1 M), and Fe^2+^/Fe^3+^ (1:2 M), which facilitated an entrapment of different types of iron ions into the casein matrix; one contained only Fe^2+^ ions, an other only Fe^3+^ ions, and one had both Fe^3+^ and Fe^2+^ ions. Afterward, these particles were subjected to precipitation of iron oxide in alkaline medium (Gupta et al. 2014a, b). Thus, it can be hypothesized that the CNPs containing only Fe^3+^ and Fe^2+^ ions lead to the formation of maghemite (c-Fe_2_O_3_) and hematite (a-Fe_2_O_3_) oxides of iron, whereas CNPs containing both Fe^2+^ and Fe^3+^ ions in the 1:2 molar ratio preferred the formation of magnetite (Fe_3_O_4_). The mechanistic aspects of iron oxide formation are represented by the following chemical equations. The formation of hematite or maghemite from ferrous ions inside a polymer matrix via formation of iron hydroxide may be shown as

8 $${\text{Fe}}^{2+}+2\text{OH}-\to \text{Fe}\;{\left(\text{OH}\right)}_{2}$$

9 $$2\text{Fe}\;{\left(\text{OH}\right)}_{2}\to {\text{Fe}}_{2}{\text{O}}_{3}+3{\text{H}}_{2}\text{O}.$$

In case of oxidation, the ferrous hydroxide formed can also transform into ferric hydroxide. The ferrous and ferric forms of hydroxides lead to the formation of magnetite as follows:

10 $${\text{Fe}}^{3+}+3\text{OH}-\to \text{Fe}{\left(\text{OH}\right)}_{3}$$

11 $$\text{Fe}\;{\left(\text{OH}\right)}_{2}+2\text{Fe}\;{\left(\text{OH}\right)}_{3}\to {\text{Fe}}_{3}{\text{O}}_{4}+4{\text{H}}_{2}\text{O}$$

### FTIR spectra

**Fig. 2:**
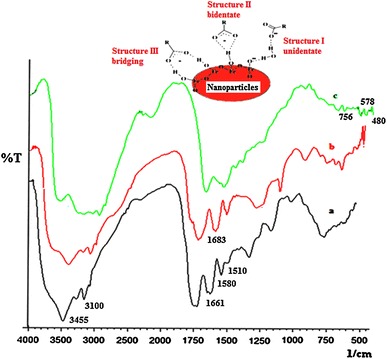
FTIR spectra of **a** native casein, **b** CNPs, and **c** CCIONPs

The FTIR spectra of casein, casein nanoparticles, and CCIONPs are shown in Fig. 2a, b, and c, respectively. Figure 2a shows absorption bands at 3,455, 3,100, 1,661, 1,530, and 1,235 cm^−1^ which can be explained as follows: In the case of native casein, the amide A band at 3,455 cm^−1^ and amide B at 3,100 cm^−1^ are observed, which are originated as a result of Fermi resonance between the first overtone of amide II, and the N–H stretching vibrations are specific for casein. Amide I and amide II bands are two major bands of the infrared spectrum of casein. The observed intense band for amide I comes at around 1,661 cm^−1^ and is mainly associated with the C = O stretching vibration and is directly related to the backbone conformation and hydrogen bonding. The amide II band is obtained in the 1,510 and 1,580 cm^−1^ region (Gu et al. 2010). Amide II results from the N–H bending vibration and from the C–N stretching vibration. The obtained bands at 1,661 and 1,531 cm^−1^ for the amide I and amide II, respectively, also confirm the alpha helical structure of casein protein (Luginbuhl 2002).

Casein also exhibited another characteristic band at 1,415 cm^−1^, attributed to the carboxylate group (O–C–O). Some characteristic peaks of iron oxide are also present. In Fig. 2b, a band observed at 1,683 cm^−1^ is due to C=N stretching, which confirms the presence of crosslinking between casein and glutaraldehyde. Appearance of a peak in Fig. 2c around 480–450 cm^−1^, 578 and 756 cm^−1^ may be attributed to Fe–O bonds of magnetite, which are characteristic peaks of iron oxide (e.g., polyhedral Fe^3+^–O^2−^) stretching vibrations of iron oxide, which confirms the coating of iron oxide by the casein (Wu and Lien 2008).

### SEM analysis

**Fig. 3:**
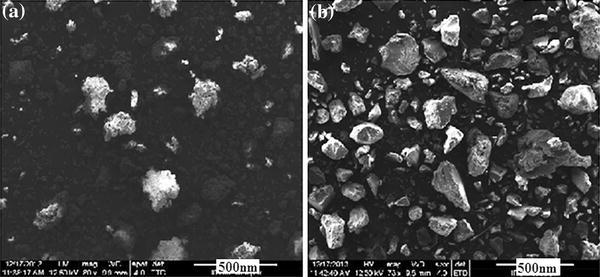
SEM images of **a** CNPs, and **b** CCIONPs

SEM images of CNPs and CCIONPs are shown in Fig. 3a and b, illustrating the clear, irregular, non-smooth morphology of CNPs and formation of iron oxide in the casein networks. The coating by casein provides for larger particle sizes due to the formation of the coating layers on the surfaces of iron oxide. It may be inferred that iron oxides are assembled or attached inside the biopolymer networks and on the surface. Loading of iron oxide inside the network affects its morphology and structural integrity. These biopolymer networks may be considered as nanoreactors to construct or assemble iron oxide. The results may be attributable to a combined contribution of a Fe–O^−^ coordination bond on the surface, steric effect, and a compartment effect of the network structures of casein, which limit the growth of iron oxide, and thus play an important role in the process of the formation of iron oxide aggregates (Murthy et al. 2008). It is also observed that the degree of aggregation is small in CCIONPs in comparison to CNPs with nonuniform size distribution in the range. The SEM image also reveals that the size of nanoparticles is not uniform and varies in the range 50 to 300 nm. The particle size distribution curve of prepared nanoparticles is shown in Fig. 6, which implies that the dimensions of nanoparticles vary in the range 50 to 300 nm, confirming the SEM observation.

### TEM analysis

**Fig. 4:**
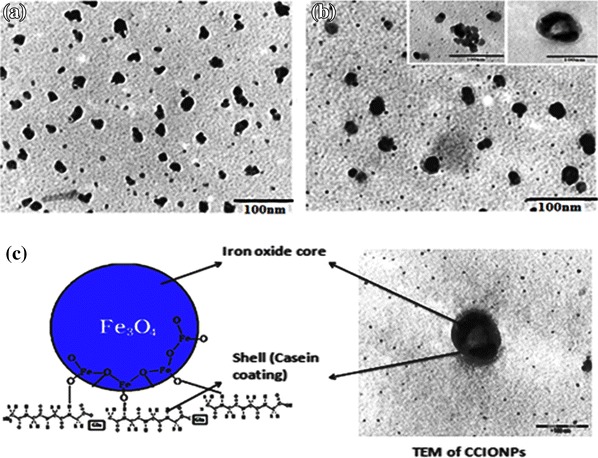
TEM images of **a** CNPs and **b** CCIONPs. **c** Images of CCIONPs showing core shell structures

In order to ascertain the size of the CNPs and CCIONPs, TEM studies were performed. The TEM image of native CNPs and CCIONPs and core shell structure of CCIONPs are shown in Fig. 4a, b, and c, respectively, which indicate that an average size of the casein nanoparticles falls in the range of 48.3 ± 0.91 nm; for CCIONPs it is found in the range of 73.9 ± 0.36 nm. The figure shows that the nanoparticles are present in aggregated form which could be attributed to the reason that the nanoparticles have a natural tendency to undergo clustering due to variety of charges present on their surfaces. In the present work, casein nanoparticles bear an overall positive charge because of the cationic nature of casein and, therefore, form a cluster-like appearance as evidenced from the TEM image. In CCIONPs, similar aggregation may be attributed to casein being hydrophilic, showing a great tendency to get agglomerated with iron oxide nanoparticles.

On comparing the TEM image of CCIONPs with CNPs, it can be clearly demonstrated that CCIONPs comprise of core/shell structure with the homogenous incorporation of magnetite as a core of CCIONPs. The magnetic nanoparticles are homogeneously covered by the non magnetic layer of casein. In Fig. 4b, a high resolution TEM image of CCIONPs is shown, which also confirms the presence of iron oxide nanoparticles in the polymer matrix. By analyzing the TEM image, it is also observed that aggregated CCIONPs have a non-smooth surface morphology. It also reflects the dark appearance and an increment in particle size confirmed the in situ formation and impregnation of iron oxide nanoparticles as the core of the nanoparticle matrix. Core/shell structure appeared in Fig. 4c, as a concentrated core and a lighter shell, in which magnetite nanoparticles appeared darker and well dispersed in the whole matrix of nanoparticle (Gupta et al. 2014a, b).

### ED analysis

**Fig. 5:**
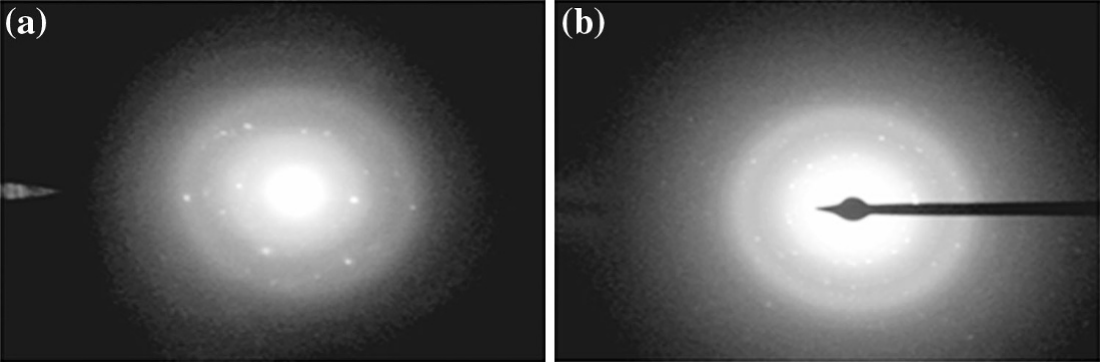
ED images of CNPs and CCIONPs

The crystalline nature of casein and CCIONPs was investigated using ED analysis. ED of the CCIONPs gives rise to very broad and weak rings, showing the amorphous nature of the polymer matrix. Results shown in Fig. 5a and b reveal the ED pattern images; the appearance of diffraction pattern for CCIONPs confirms the crystalline nature of the nanoparticles. The diffraction pattern obtained from CNPs have two holes with (211) and (022), characteristic of complex proteins that are without sufficient structural orientation and organization.

The lack of structural organization in casein is due to the mutual attraction of the large number of polar groups that probably occur in a complex casein structure. The polar groups are apparently distributed at irregular intervals in both side chains and along main chains; hence, the structural design is not of regular geometric pattern. The ED pattern of the protein structure shows that side chains extend from the main chain at regular intervals and these serve as definite diffraction units in determining the perpendicular distance, in the same plane, between the polypeptide chains. This spacing is exceedingly varied since it seems to depend to a large extent on the amount of water held by the protein. For the majority of proteins, this value has been calculated to be from 9.5 to 10.5 Å, although some workers (Arnlund et al. 2014) have found it to vary from 10.4 to 17 Å for certain proteins, depending on the amount of water bound by the protein. In addition to this 9.8 Å spacing that is always present in diffraction patterns of proteins, another one appears, representing the perpendicular distance or thickness between planes of polypeptide chains. This value is rather uniform for all proteins, being for the majority 4.6 Å (Thorn et al. 2005). The high resolution picture clearly indicates the particles are single crystalline. The ED pattern shows (220), (311), (400), (422), (511), and (440) rings originating from the magnetite (Fe_3_O_4_) and/or maghemite (γ-Fe_2_O_3_) structures since the positions of the diffraction patterns are almost the same in both of them (Sun et al. 2004).

### DLS measurements

DLS also known as photon correlation spectroscopy (PCS), is a common technique used for the determination of particle size and size distribution in colloidal dispersions. The velocity distribution of particle movement is analyzed with DLS (Murdock et al. 2008). This is achieved by measuring the temporal dynamic fluctuations of laser light scattering intensity caused by the Brownian motion of the particles. Analysis of these intensity fluctuations enables the determination of the distribution of particle diffusion coefficients, which are converted into a size distribution using established theories. Scattering intensity depends on the size and absorption of the particles, the scattering angle, as well as on the refractive indices of both the particles and the dispersion medium.

**Fig. 6:**
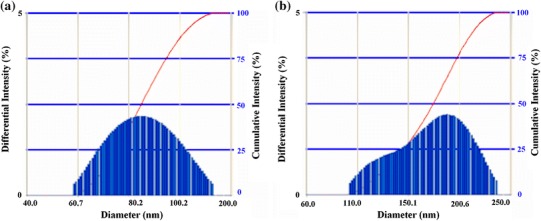
DLS images of CNPs and CCIONPs

The DLS particle size distribution plot is shown in Fig. 6, indicating the CNPs and CCIONPs have moderate size distribution with a polydispersity index of 0.566. It is reported that PDI values greater than 0.7 indicate that the sample has a very broad size distribution (Nita et al. 2011). The small polydispersity index suggests that nucleation is fast as compared to particle growth, and also that the secondary nucleation step is absent. The average size (diameter, nm) of CCIONPs is 200.5 nm, which confirms that the prepared nanoparticles lie in the nanometer range, whereas, in the case of CNPs, it is shown that they are quite lower in size than CCIONPs. Thus, it is also confirmed that the prepared particles fall in the nanometer range.

### Zeta potential measurements

When nanoparticles are administered intravenously, they are easily recognized by the body immune systems, and are then cleared by phagocytes from the circulation (Muller et al. 1993). Apart from the size of nanoparticles, their surface hydrophobicity determines the amount of blood components adsorbed, mainly proteins.

The efficiency of surface modification can be measured either by estimating the surface charge, density of the functional groups, or an increase in surface hydrophilicity. Zeta potential is frequently used to measure the surface modification of an aqueous suspension containing nanoparticles. It reflects the electrical potential of particles and is influenced by the composition of the particle and the medium in which it is dispersed. The main reason for measuring the zeta potential is to predict colloidal stability. Interactions between particles play an important role in colloidal stability. The zeta potential is a measure of the repulsive forces between particles and since most aqueous colloidal systems are stabilized by electrostatic repulsion, the larger the repulsive forces between particles, the less likely they will come closer to forming an aggregate. Nanoparticles with a zeta potential above (±) 30 mV have been shown to be stable in suspension, as the surface charge prevents aggregation of the particles. The zeta potential can also be used to determine whether a charged active material is encapsulated within the centre of the nanocapsule or adsorbed onto the surface (Mohanraj et al. 2006).

**Fig. 7:**
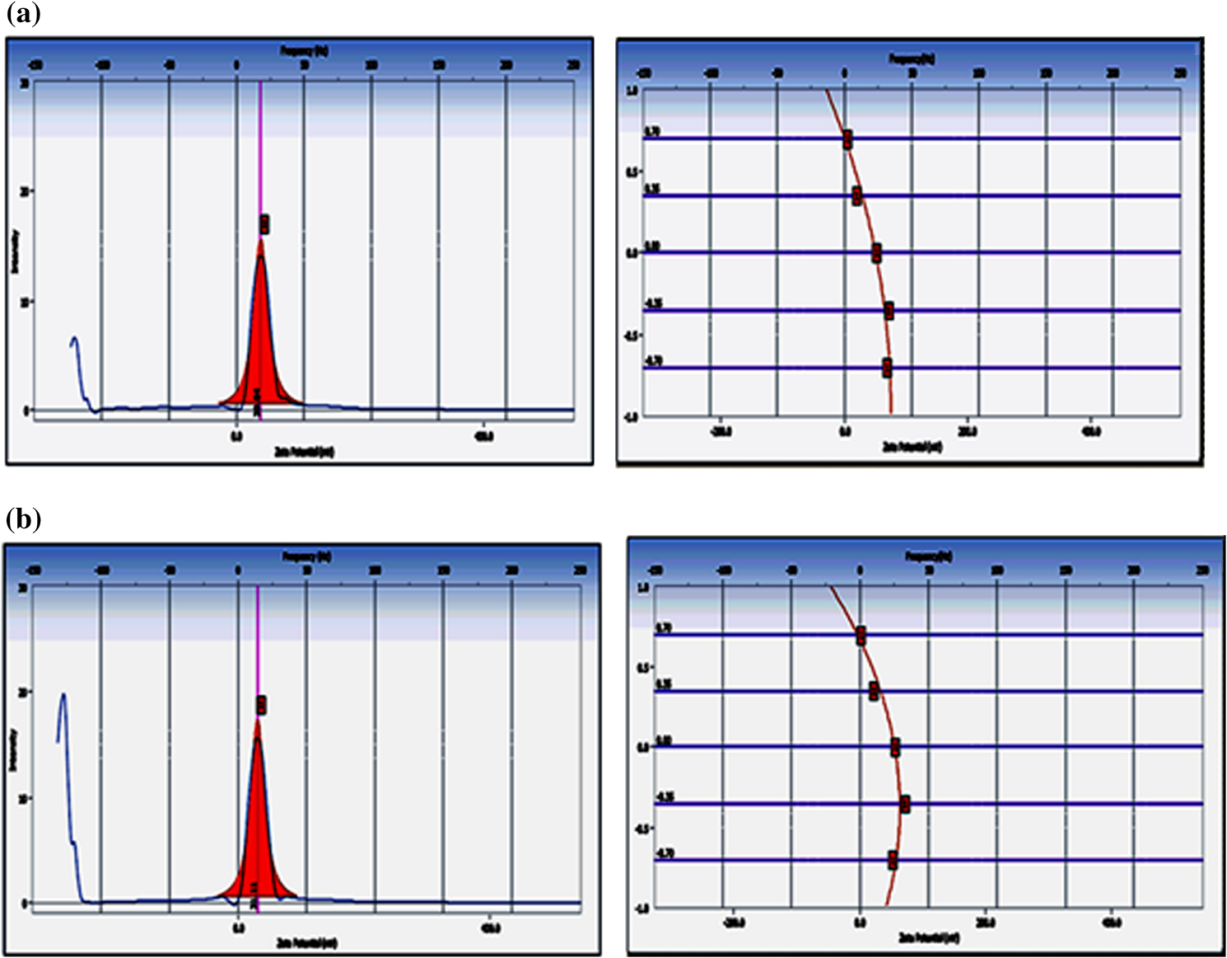
Zeta potential of CNPs and CCIONPs

In the present study, the zeta potential measurements of CNPs and CCIONPs were performed using a Zetasizer instrument. The zeta potential distribution was measured between zeta potential (mV) versus intensity (kcps) and the measurements were performed at 25 °C with a count rate of 2,272.3 kcps. The results obtained clearly indicate that zeta potential of CNPs was found to be 39.04 mV and, upon loading of iron oxide onto casein nanoparticles, a net decrease in positive potential was noticed. The zeta potential value of CCIONPs was found to be 31.06 mV. The results obtained are shown in Fig. 7a and b. The observed decrease in zeta potential of the CCIONPs may be attributed to the fact that cationic-charged centers along the casein macromolecules are held up with the negatively charged oxygen atoms of iron oxides via electrostatic attraction and, thus, resulting in a decrease in the zeta potential.

### XRD analysis

**Fig. 8:**
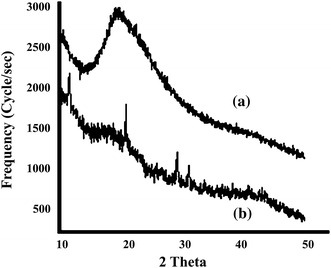
XRD images of *a* CNPs and *b* CCIONPs

The XRD spectra of prepared casein and CCIONPs are shown in Fig. 8a and b. The appearance of a broad peak in the XRD spectra of casein indicates the amorphous nature of casein nanoparticles (Fig. 8a). XRD revealed crystallinity of the CCIONPs increased with weight percentage of the magnetite nanoparticles. The XRD measurements, which were performed to determine the crystallographic structure of CCIONPs, revealed the characteristic peak of pure magnetic Fe_3_O_4_, as shown in Fig. 8b, at Bragg’s angles of 30.1, 35.5, 43.3, 57.1, and 62.7, 2*θ* with corresponding *hkl* values (220), (311), (400), (422 and 511), and (440), respectively, suggesting the impregnation of iron oxide into casein nanoparticles (Bajpai and Gupta 2010). The disappearance of the 111 peak in the CCIONP diffraction pattern is due to covering of magnetite crystallites by an amorphous biopolymer (Lee et al. 2005).

The percent crystallinity of the CCIONPs was calculated to be 96 %. Since casein is amorphous in nature and increase in the value of percent crystallinity, this suggests the impregnation of iron oxide into the matrix of casein nanoparticles. The grain size of the prepared nanoparticles was also calculated using XRD measurements. The estimated average grain size of iron-impregnated casein nanoparticles was found to be 69.3 nm.

### Raman spectral analysis

**Fig. 9:**
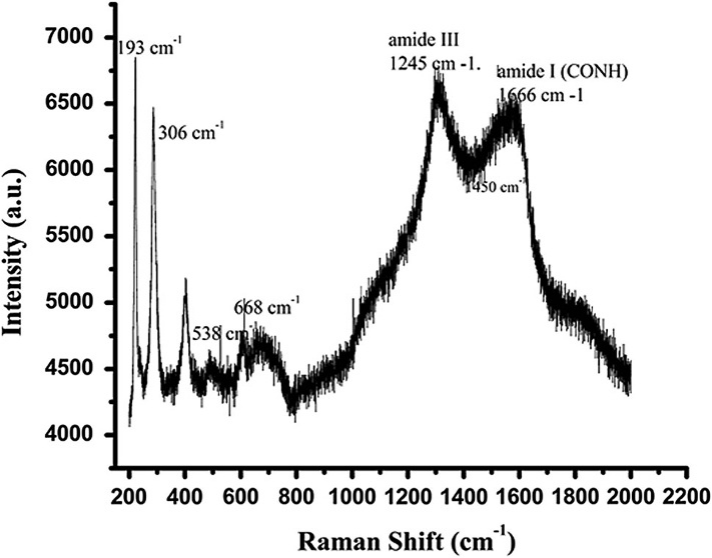
Raman spectra of CCIONPs

Raman spectroscopy allows characterization of samples without any specific preparation. Raman spectra of CCIONPs are shown in Fig. 9, showing characteristic Raman bands for casein due to amide I (CONH) at ~1,666 cm^−1^ and amide III bands at ~1,245 cm^−1^. Between these Raman bands, an intense feature is observed at 1,450 cm^−1^, which is attributed to the CH_2_ scissoring mode. In the Raman spectra, weaker peaks observed at 193, 306, and 538 cm^−1^ confirm the presence of iron oxide in the form of magnetite. An additional strong peak is also observed at 668 cm^−1^. For correct assignment of samples, a combined Raman data key can be used as follows (Chourpa et al. 2005).

(i) Fe_3_O_4_:193 (weak), 306 (weak), 538 (weak), 668 (strong),
(ii) gFe_2_O_3_:350 (strong), 500 (strong), 700 (strong); and
(iii) aFe_2_O_3_: 225 (strong), 247 (weak), 299 (strong), 412 (strong), 497 (weak), 613 (medium).

### XPS analysis

**Fig. 10:**
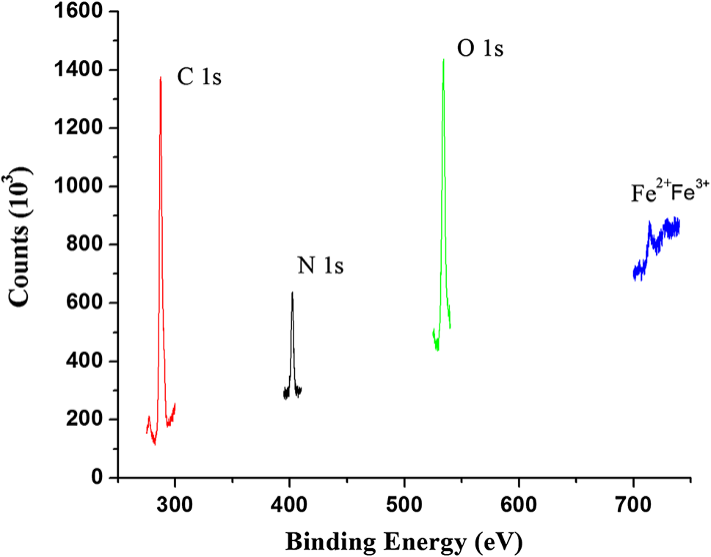
XPS of CCIONPs

In this study, XPS analyses were done to monitor the iron oxide deposition in the cross-linked CCIONPs. The spectra of the cross-liked CCIONPs show the C 1*s*, O 1*s*, and N 1*s* core-level peaks (Fig. 10). However, after impregnation of iron oxide, the spectrum exhibits two more peaks associated with Fe^2+^ and Fe^3+^, due to the iron oxide deposition. Peak assignment is based on characteristic binding energies from the literature (Shen et al. 2004: Beamson et al. 1992). Moreover, the O 1*s* core-level spectra of the cross-linked CCIONPs were fitted using two peaks at 532.3 and 534 eV. The first one is associated with the binding energy of the [C = O] in the imide group and carboxylic acid group, while the second one is associated with the binding energy of the OH in the carboxylic acid group. The absence of the peak at 287.2 eV, associated with the binding energy of carboxylic acid groups, is accompanied by an increase in the intensity of the peak at 285.9 eV due to the contribution of the carboxylate species in the crosslinked biopolymer (Alexander et al. 2001). These groups were also observed in the FTIR analysis. The O 1*s* core-level spectrum from the resulting composite was fitted to peaks at 530.2 eV (g-Fe_2_O_3_) and 531.4 eV (a-FeOOH). Moreover, the spectrum displays two peaks associated with the iron oxide in the composite, which are in good agreement with the magnetite, at 715.3 and 725.4 eV for Fe^2+^ and Fe^3+^ ions.

XPS provided elemental information of surface composition of CCIONPs after Fe_3_O_4_ loading. There were C, N, O, and Fe elements in the magnetic CCIONPs, further proving that Fe_3_O_4_ nanoparticles had been synthesized in situ in the CCIONPs (Xuan et al. 2007). The different oxidation state of the iron in these nanoparticles can also be detected and distinguished from each other by XPS.

### Mossbauer analysis

**Fig. 11:**
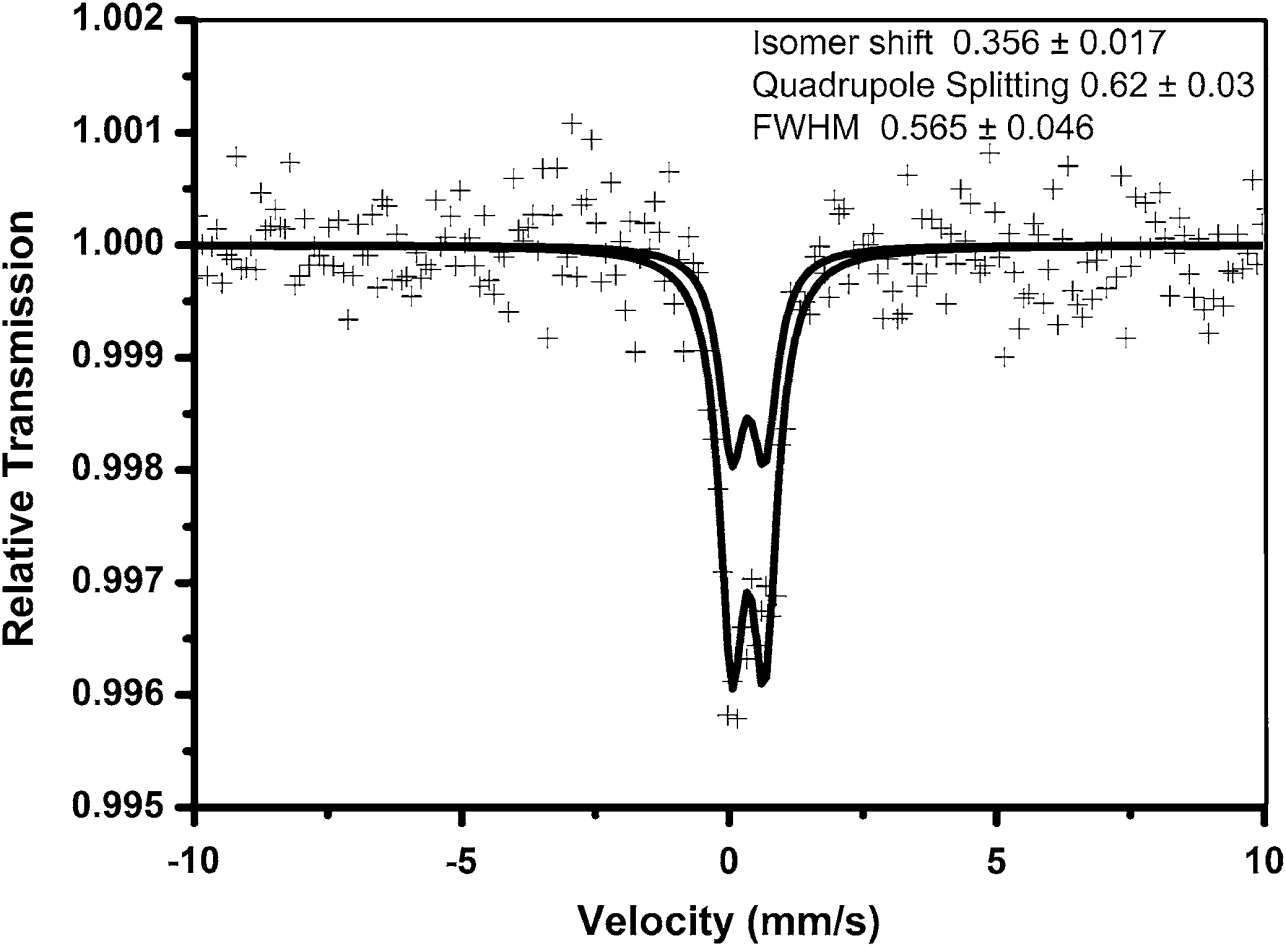
Mossbauer spectra of CCIONPs

Mossbauer spectroscopy is a very effective technique for studying individual iron oxides and iron oxide in the casein matrix, including amorphous and superparamagnetic nanoparticles. The Mossbauer spectra are quite sensitive to the local environment of the iron atoms in the crystal lattice. The hyperfine parameters, isomer shift, quadruple splitting, quadruple shift, and magnetic splitting provide information about the electronic density and its symmetry and also about the magnetic properties of the Fe Mossbauer probe nucleus (Brent Fultz 2011). Valuable information may also be obtained from the widths of the spectral lines, their relative intensities, their asymmetries and also from the temperature dependence of hyperfine parameters. From the Mossbauer parameters, the information applicable to Fe_2_O_3_ studies can be obtained, such as valence state, number, and identification of non equivalent iron positions in a crystal lattice, type of coordination of iron in its individual positions, level of ordering and stoichiometry, cation substitution, magnetic ordering, and magnetic transition temperature (Siddique et al. 2010). The Mossbauer data presented in this paper indicate magnetic behavior of prepared nanoparticles. At room temperature, the sample is in a superparamagnetic state (below their Neel temperature), characterized, for instance, by reversible sigmoid magnetization curves. Mossbauer spectra of magnetic casein nanoparticles obtained at 300 K with external magnetic field up to 5.5 T perpendicular to the direction of the γ-ray is shown in Fig. 11.

Figure 11 obtained shows a doublet, which is an indication of superparamagnetic behavior of prepared nanoparticles, with no evidence of magnetic order (absence of magnetic sextet pattern). The fitted parameters of the spectrum are an isomer shift (IS) of 0.356 ± 0.017 mm/s, quadruple splitting (QS) of 0.62 ± 0.03 mm/s, and an FWHM of 0.565 ± 0.046.

### VSM (Vibrating sample magnetometer)

**Fig. 12:**
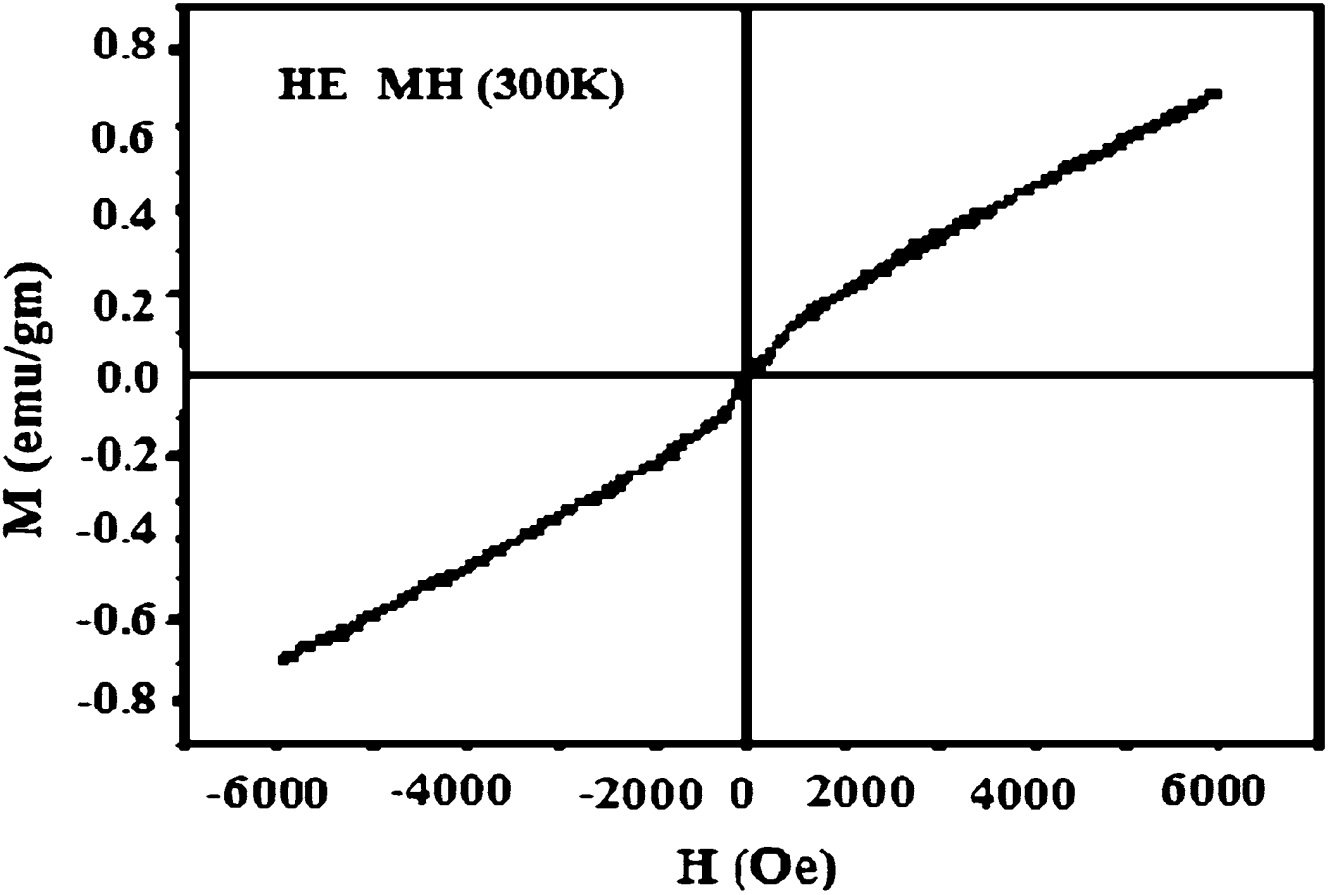
VSM of CCIONPs

The magnetization versus magnetic field plot (M-H magnetization curve and hysteresis loop) at 300 K for the CCIONPs was measured over the range of an applied field between −6,000 and +6,000 Oe, with a sensitivity of 0.1 emu g^−1^(Fig. 12). Measurements have shown that the saturation magnetization value is around 64 emu g^−1^, and the hysteresis is very weak. The value obtained is lower than the reported value of 92–100 emu g^−1^ for magnetite nanoparticles (Chia et al. 2006). It is also observed that the magnetization decreases from the plateau value and tends to reach zero as the magnetic field is gradually removed. This clearly indicates that they have neither coercivity nor remanent magnetization and, thus, proves their superparamagnetic nature. The reason for the superparamagnetic nature of the nanoparticles is essentially due to the very small size of incorporated iron oxide particles, which favors the redispersion of magnetic nanoparticles after the external magnetic field is removed.

The obtained reduction in saturation magnetization of CCIONPs particles could be attributed to the presence of a non-magnetic layer on the surface of the particles, charge distribution, super-paramagnetic relaxation, blocking temperature, and spin effect because of the ultrafine nature of particles. It is well known that above the blocking temperature (*T*_B_), superparamagnetic nanoparticles become thermally unstable and the magnetization value decreases exponentially as *MV*/*kT* becomes larger than 1 (Jiles et al. 2005), where *M* is the magnitude of magnetization, *V* is the particle volume, *k* is the Boltzmann constant, and *T* is the absolute temperature. When the particles are chemically coated, the blocking temperature is suppressed to a lower temperature. In the absence of any coating, due to the increase in the large ratio of surface area to volume, the attractive force between the nanoparticles increases and agglomeration of the nanoparticles takes place. These agglomerated nanoparticles act as a cluster, resulting in an increase in blocking temperature. Surface coated particles are more freely aligned with the external field than the uncoated nanoparticles. The total effective magnetic moment of such coated particles is found to decrease, which is most likely due to a non- collinear spin structure originating from the pinning of surface spins and coated polymer at the interface of the nanoparticles (Savva et al. 2014; Sun et al. 2007).

### In vitro cytotoxicy test

**Fig. 13:**
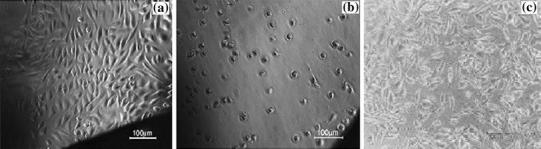
Microscopic images showing an L-929 cell around **a** negative control **b** positive control and **c** CCIONPs

The cytocompatibility of CCIONPs was judged by in vitro cytotoxicity testing as performed on a L-929 fibroblast by the extract method. The cytotoxicity reactivity of test and control samples was evaluated under an inverted phase contrast microscope. As per ISO 10993-5, a numerical grade greater than 2 is considered cytotoxic. The cytotoxicity reactivity was graded based on the zone of lysis, vacuolization, detachment, and membrane disintegration; 0, 1, 2, 3, and 4 represent none, slight mild, moderate, and severe, respectively. The negative control was graded 0, showing no reactivity, while the positive control displayed severe reactivity with a grade of 4. The quantitative evaluation of reactivity for negative and positive controls and the test sample are summarized in Table 1, while microscopic observation are depicted in Fig. 13a, b, and c, respectively. It was found that the test sample, i.e., CCIONPs, exhibited no reactivity with a grade of 0 on L-929 fibroblast cells after 24 h of contact, implying the culture condition has discrete intracytoplasmic granules, no cell lysis, and no reduction of cell growth, as shown in Fig. 13c. The observed lack of cytotoxicity to fibroblasts cells may be attributed to the non-toxic and biocompatible nature of the casein. This proves the biocompatible nature of CCIONPs.

**Table 1:** Quantitative evaluation of in vitro cytotoxic reactivity of various samples

S. no	Sample	Grade	Reactivity
1.	Negative control	0	None
2.	Positive control	4	Severe
3.	IOICNPs	0	None

### Conclusions

In the present study, casein nanoparticles were synthesized using an emulsion crosslinking method. For the impregnation of iron oxide, an in situ co-precipitation by divalent and trivalent iron from the respective hydrated salts in an alkaline medium was performed in a (Fe^2+^/Fe^3+^) molar ratio of 1:2. The main focus was to produce CCIONPs having a magnetite form of iron oxide due to its better biocompatibility and lower toxicity than other forms of iron oxides, such as hematite or maghemite. Various techniques, like FTIR, XRD, Raman spectroscopy, VSM, and Mossbauer spectroscopy, were used to characterize the prepared nanoparticles. Peaks around 1,683 and 540 cm^−1^ confirms the crosslinking of casein with glutaraldehyde and impregnation of iron oxide into the polymer matrix, respectively. Other supporting techniques, such as XRD, Raman spectroscopy, VSM, and Mossbauer, were used, confirming the presence of iron oxide in the nanoparticles.

TEM studies also confirmed the core shell type morphology and that the average size of the casein nanoparticles is 40–60 nm; for CCIONPs, the average size was 73.9 ±0.36 nm. TEM analysis showed that the nanoparticles were well dispersed in the polymer matrix. They retained their superparamagnetic nature even when coated by casein. The high loading of magnetic nanoparticles resulted in relatively high saturation magnetizations of the nanocomposites, up to 64 emu g^−1^. The zeta potential of casein nanoparticles was found to be 39.04 mV and, upon loading of iron oxide onto casein nanoparticles, a net decrease in positive potential to 31.06 mV was noticed, confirming the electrostatic interaction between cationic charged centers along the casein with the negatively charged oxygen atoms of iron oxides. The estimated average grain size of iron-impregnated casein nanoparticles was found to be 69.3 nm by using XRD analysis. In vitro cytotoxicity testing confirms the biocompatible nature of prepared CCIONPs with no cytotoxic effect on L-929 fibroblast cells. Thus, it can be concluded that new biocompatible superparamagnetic nanoparticles have been designed as potential nanocarriers that can be used in magnetic drug targeting.

## References

[CR1] Alexander MR, Beamson G, Blomfield CJ, Leggett G, Duc TM (2001). Interaction of carboxylic acids with the oxyhydroxide surface of aluminium: poly(acrylic acid), acetic acid and propionic acid on pseudoboehmite. J Electron Spectrosc Relat Phenom.

[CR2] Arnlund D, Johanssen LC, Wickstrand C, Barty A, Williams GJ, Malmerberg E, Davidssen J, Milathianaki D, Deponte DP, Shoeman RL, Wang D, James D, Katona G, Westenhoff S, While TA, Aquila A, Bari S, Berntsen P, Bagon M, Vandriel TB, Bruce R (2014). Visualizing a protein quake wth time resolved X-ray scattering at a free electron laser. Nat Methods.

[CR3] Bajpai AK, Gupta R (2010). Magnetically guided release of ciprofloxacin from superparamagnetic polymer nanocomposites. J Biomater Sci.

[CR4] Beamson G, Briggs D (1992). High resolution XPS of organic polymers—the Scienta ESCA 300 Database.

[CR5] Bouchemal K, Briançon S, Perrier E, Fessi H (2004). Nano-emulsion formulation using spontaneous emulsification: solvent, oil and surfactant optimization. Int J Pharm.

[CR7] Chia CH, Zakaria S, Ahmad S, Abdullah MH, Mohd Jani S (2006). Preparation of magnetic paper from kenaf: lumen loading and in situ synthesis method. Am Appl Sci.

[CR8] Chomoucka J, Drbohlavova J, Huskab D, Adamb V, Kizekb R, Hubaleka J (2010). Magnetic nanoparticles and targeted drug delivering. Pharmacol Res.

[CR9] Chourpa I, Douziech-Eyrolles L, Ngaboni-Okassa L, Fouquenet JF, Cohen-Jonathan S, Souce M, Marchais H, Dubois P (2005). Molecular composition of iron oxide nanoparticles, precursors for magnetic drug targeting, ascharacterized by confocal Raman microspectroscopy. Analyst.

[CR10] Dalgleish DG (1998). Casein micelles as colloids: surface structures and stabilities. J Dairy Sci.

[CR11] Fox PF, Fox PF, McSweeney PLH (2003). Milk proteins: general and historical aspects. Part A: advanced dairy chemistry-1 proteins.

[CR12] Fultz B, Kaufmann E (2011). Mössbauer spectrometry. Characterization of materials.

[CR14] Gu FL, Kim JM, Abbas S, Zhang XM, Xia SQ, Chen ZX (2010). Structure and antioxidant activity of high molecular weight Maillard reaction products from casein–glucose. Food Chem.

[CR15] Gupta MK, Bajpai J, Bajpai AK (2014). Preparation and characterizations of superparamagnetic ironoxide-embedded poly(2-hydroxyethyl methacrylate) nanocarriers. J Appl Polym Sci.

[CR16] Gupta MK, Bajpai J, Bajpai AK (2014). The biocompatibility and water uptake behavior of superparamagnetic poly (2-Hydroxyethylmethacrylate)—magnetite nanocomposites as possible nanocarriers for magnetically mediated drug delivery system. J Poly Res.

[CR17] Gyergyek S, Huski M, Makovec D, Drofenik M (2008). Superparamagnetic nanocomposites of iron oxide in a polymethylmethacrylate matrix synthesized by in situ polymerization. Colloids Surf A Physicochem Eng Aspects.

[CR18] Hoo CM, Starostin N, West P, Mecartney ML (2008). A comparison of atomic force microscopy (AFM) and dynamic light scattering (DLS) methods to characterize nanoparticle size distributions. J Nanopart Res.

[CR19] Indira TK, Lakhsmi PK (2010). Magnetic Nanoparticles- A Review. Int Pharm Sci Nanotechnol.

[CR20] Ismail H, Irani M, Ahmad Z (2013). Starch-based hydrogels: presentatus and applications. Int J Polym Mater Polym Biomater.

[CR21] Jahanshahi M, Babaei Z (2008). Protein nanoparticle: a unique system as drug delivery vehicles. Afr J Biotechnol.

[CR22] Jiles D (2005) Introduction to Magnetism and Magnetic Materials. In: Jonathan, S, Souce M, Marchais H, Dubois P (eds) Analyst. Chapman and Hall, 130 pp 1395–1403 10.1039/b419004a 16172665

[CR23] Jung KW, Oh JMP (2011). Iron oxide-based superparamagnetic polymeric nanomaterials: design, preparation, and biomedical application. Prog Poly Sci.

[CR24] Kim DK, Zhang Y, Voit W, Rao KV, Muhammed M (2001). Synthesis and characterization of surfactant-coated superparamagnetic monodispersed iron oxide nanoparticles. J Magn Magn Mater.

[CR25] Lee SJ, Jeong JR, Shin SC, Kim JC, Chang YH, Lee KH, Kim JD (2005). Magnetic enhancement of iron oxide nanoparticles encapsulated with poly(d, l-latide-co-glycolide). Colloids Surf A.

[CR27] Likhitkar S, Bajpai AK (2013). Investigation of magnetically enhanced swelling behavior of superparamagnetic starch nanoparticles. Bull Mater Sci.

[CR28] Lu AH, Salabas EL, Schuth F (2007). Magnetic nanoparticles: synthesis, protection, functionalization, and application. Angew Chem Int Ed.

[CR29] Luginbuhl W (2002). Evaluation of designed calibration samples for casein calibration in fourier transform infrared analysis of milk. Lebensm-Wiss u-Technol.

[CR30] Migneault I, Dartiguenave C, Bertrand MJ, Waldron KC (2004). Glutaraldehyde: behavior in aqueous solution, reaction with proteins, and application to enzyme crosslinking. Biotechniques.

[CR31] Mohanraj VJ, Chen Y (2006). Nanoparticles—a review. Trop J Pharm Res.

[CR32] Müller RH, Wallis KH (1993). Surface modification of i.v. injectable biodegradable nanoparticles with poloxamer polymers and poloxamine. Int J Pharm.

[CR33] Murdock RC, Braydich-Stolle L, Schrand AM, Schlager JJ, Hussain SM (2008). Characterization of nanomaterial dispersion in solution prior to in vitro exposure using dynamic light scattering technique. Toxicol Sci.

[CR34] Murthy PSK, Mohan YM, Varaprasad K, Sreedhar B, Raju KM (2008). First successful design of semi-IPN hydrogel–silver nanocomposites: a facile approach for antibacterial application. J Colloid Interface Sci.

[CR35] Nita LE, Chiriac AP, Nistor M, Stoica I (2011). Biomaterials based on 2-hydroxyethyl methacrylate: the influence of the initiator type. Rev Roum Chim.

[CR36] Oh JK, Jong Myung Park M (2011). Iron oxide-based superparamagnetic polymeric nanomaterials: design, preparation, and biomedical application. Prog Polym Sci.

[CR37] Phadungath C (2005). Casein micelle structure: a concise review. J Sci Technol.

[CR38] Sarkar S, Guibal E, Quignard F, SenGupta AK (2012). Polymer-supported metals and metal oxide nanoparticles: synthesis, characterization, and applications. J Nanopart Res.

[CR13] Savva I, Constantinou D, Marinica O, Vasile E, Vekas L, Krasia-Christoforou T (2014). Fabrication and characterization of superparamagnetic poly(vinyl pyrrolidone)/poly(L-lactide)/Fe_3_O_4_ electrospun membranes. J Magn Magn Mater.

[CR40] Shen G, Anand MFG, Levicky R (2004). X-ray photoelectron spectroscopy and infrared spectroscopy study of maleimide-activated supports for immobilization of oligodeoxyribonucleotides. Nucleic Acids Res.

[CR41] Shi R, Zhu A, Chen D, Jiang X, Xu X, Zhang L, Tian W (2010). In vitro degradation of starch/PVA films and biocompatibility evaluation. J Appl Polym Sci.

[CR42] Siddique M, Ahmed E, Butt NM (2010). Particle size effect on Mössbauer parameters in γ-Fe_2_O_3_ nanoparticles. Physical B.

[CR43] Singh A, Bajpai J, Bajpai AK (2014). Investigation of magnetically controlled water intake behavior of Iron Oxide Impregnated Superparamagnetic Casein Nanoparticles (IOICNPs). J Nanobiotechnol.

[CR44] Sun S, Zeng H, Robinson DB, Raoux S, Rice PM, Wang SX, Li G (2004). Monodisperse MFe2O4 (M = Fe Co, Mn) nanoparticles. J Am Chem Soc.

[CR45] Sun J, Zhou S, Hou P, Yang Y, Weng J, Li X, Li M (2007). Synthesis and characterization of biocompatible Fe_3_O_4_ nanoparticles. J Biomed Mater Res Part A.

[CR46] Thompson A, Boland MJ, Singh H (2009) Milk proteins, from expression to food, 1 edn. Elsevier, Academic Press, Taylor SL., Amsterdam

[CR47] Thorn DC, Meehan S, Sunde M, Rekas A, Gras SL, Macphee CE, Dobson CM, Wilson MR, Carvev JA (2005). Amyloid fibril formation by bovine milk kappa-casein and its inhibition by the molecular chaperones alpha S- and beta- casein. Biochemistry.

[CR48] Wan J, Tang G, Qian Y (2007). Room temperature synthesis of single-crystal Fe_3_O_3_ nanoparticles with superparamagnetic property. App Phys A.

[CR49] Wu TM, Lien YH (2008). Preparation and characterization of thermosensitive polymers grafted on to silica coated iron oxide nanoparticles. J Colloid Interface Sci.

[CR50] Xuan SH, Fang QL, Hao LY, Jiang WQ, Gong XL, Hu Y, Chen ZY (2007). Fabrication of spindle Fe_2_O_3_@polypyrrole core/shell particles by surface-modified hematite templating and conversion to spindle polypyrrole capsules and carbon capsules. J Colloid Interface Sci.

[CR51] Yang YY, Wang Y, Powell R, Chan P (2006). Polymeric core-shell nanoparticles for therapeutics. Clin Exp Pharmacol P.

[CR52] Zou X, Hovmöller S, Oleynikov P (2011) Electron crystallography: electron microscopy and electron diffraction (International Union of Crystallography: Iucr Texts on Crystallography)

